# Successful and failed mini-implants: microbiological evaluation and quantification of bacterial endotoxin

**DOI:** 10.1590/1678-7757-2017-0631

**Published:** 2018-06-25

**Authors:** Marcela Cristina Damião ANDRUCIOLI, Mírian Aiko Nakane MATSUMOTO, Maria Conceição Pereira SARAIVA, Magda FERES, Luciene Cristina de FIGUEIREDO, Carlos Artério SORGI, Lucia Helena FACCIOLI, Raquel Assed Bezerra da SILVA, Lea Assed Bezerra da SILVA, Paulo NELSON-FILHO

**Affiliations:** 1 Universidade de São Paulo, Faculdade de Odontologia de Ribeirão Preto, Departamento de Clínica Infantil, Ribeirão Preto, São Paulo, Brasil.; 2 Universidade de Guarulhos, Divisão de Pesquisa Odontológica, Departamento de Periodontia, Guarulhos, São Paulo, Brasil.; 3 Universidade de São Paulo, Faculdade de Ciências Farmacêuticas, Departamento de Análises Clínicas, Toxicológicas e Bromatológicas, Ribeirão Preto, São Paulo, Brasil.

**Keywords:** Orthodontic anchorage procedures, Microbiology, Gram-negative bacteria

## Abstract

**Objectives:**

Using two groups of mini-implants (successful and failed) the objectives of this *in vivo* study were: to evaluate the microbial contamination by the checkerboard DNA-DNA hybridization technique and to quantify the bacterial endotoxin by the limulus amebocyte lysate assay.

**Material and Methods:**

The 15 successful and 10 failed mini-implants (1.6 mm diameter × 7.0 or 9.0 mm long), placed in the maxilla and/or mandible, were obtained from 15 patients undergoing orthodontic treatment. Data were analyzed statistically by the Wilcoxon rank-sum test using the SAS software (a=0.05).

**Results:**

All 40 microbial species were detected in both groups of mini-implants, with different frequencies. No differences were observed between the groups with respect to microbial complexes (blue, purple, yellow, green, orange, red and other species) and endotoxin quantification (p>0.05).

**Conclusion:**

Neither microbial contamination nor endotoxin quantification was determinant for the early loss of stability of the mini-implants.

## Introduction

In the last decades, mini-implants have been widely used in Orthodontics as temporary bone anchorage devices to provide greater mechanical control with no need of patient cooperation^1^. According to the literature, mini-implants have a high clinical success rate (>80%)^2^ ^,^ ^3^. However, there are reports of early failure involving loss of stability during the treatment^4^. Different variables may influence the success rate and they may relate to: characteristics of the patient; characteristics, location and cleaning of the mini-implant; surgical placement technique; and orthodontic mechanics^2^ ^,^ ^5^. Mini-implants are placed transgingivally and are therefore directly accessible to all types of microorganisms in the oral cavity, specially bacteria associated with periodontitis and periimplantitis. These bacteria can penetrate the mini-implant, causing infection of soft and mineralized tissues, especially in patients with poor oral hygiene^6^. The colonization of mini-implant surfaces by pathogenic bacteria has been referred to as one of the contributing factors for the failure of these devices, but this possibility should be further investigated^7^.

It is known that the periodontopathogenic microbiota predominantly consists of anaerobes^8^, mostly Gram-negative microorganisms^9^, which have endotoxin (also known as LPS due to its lipopolysaccharide nature) in their cell wall^10^. Endotoxin is released after the death or multiplication of these bacteria and represents a major virulence factor by acting as a potent stimulus for proinflammatory cytokine expression and amplification of the host immune response^11^, resulting in the development of inflammatory reaction and bone resorption^10^ ^-^ ^12^.

Microbial contamination and persistent peri-implant inflammation are two potential causes to be considered and thus microbiological analyses and detection of endotoxin on mini-implants with and without stability should be performed. This knowledge could lead to the development of strategies that can guarantee the long-term success of mini-implants.

Using two groups of mini-implants – stable (successful) and unstable (failed) – the objectives of this *in vivo* study were: 1) to evaluate the microbial contamination, using DNA probes for 40 bacterial species, by the checkerboard DNA-DNA hybridization (CDDH) biomolecular technique and 2) to quantify the bacterial endotoxin in both groups of mini-implants by the limulus amebocyte lysate assay.

## Material and methods

After the research protocol was approved by the institutional Ethics Committee (Process #19866013.0.0000.5419), the patients or their legal representatives signed a written informed consent form for participation. The Declaration of Helsinki guidelines were followed in this investigation.

Initially, sample calculation was performed using SAS Power software and Sample Size 3.1 software for the Wilcoxon two-sample test and a test power between 0.6 and 0.887, respectively, with differences of medians between groups of 200,000 to 400,000 bacteria.

A total of 15 patients, aged between 11 and 49 years, of both genders, who were under corrective orthodontic treatment with fixed appliances at the Orthodontics Clinic were enrolled in the study within a period of 12 months. The participants had good general and oral health, were nonsmokers and had not used antibiotics or anti-inflammatory drugs within 3 months before the mini-implant removal. Two groups of mini-implants were obtained: 15 well-fixed mini-implants (successful), which were removed after completion of orthodontic mechanics or at the end of the treatment, and 10 unstable mini-implants, which were removed early because of excessive mobility and became loose before the desired tooth movement could be achieved (failed).

The mini-implants (1.6 mm diameter x 7.0 or 9.0 mm long; Neodent; Curitiba, Paraná, Brazil) were placed in the maxilla and/or mandible. All mini-implants were placed and removed by the same experient surgeon, using the same surgical technique, presented no contact with adjacent tooth roots and all devices presented primary stability immediately after placement. All patients received the same postsurgical instructions to clean the peri-implant area with a soft-bristle toothbrush during toothbrushing and rinse the mouth with an antiseptic solution once a day during the period of use of the mini-implant. The mean time of permanence in the mouth was 26.1 months for successful mini-implants and 6.7 months for failed mini-implants.

At the removal, the mini-implants were stored individually in nonpyrogenic 1.5 mL Eppendorf^®^ Safe-Lock microcentrifuge tubes (Merck, Darmstadt, Hessen, Germany) containing 200 µL of pyrogen-free water. Each tube was labeled and vigorously agitated in a shaker (Mixtron; Toptronix, São Paulo, São Paulo, Brazil) for 30 seconds for desorption of the material adhered to mini-implant surfaces. From the 200 µL of bacterial suspension, 150 µL were centrifuged at 4,000 g for 12 minutes to eliminate the supernatant. The pellet was resuspended in 150 µL TE (Tris EDTA) buffer and 100 µL 0.5 M NaOH and stored frozen at **−**20°C for further processing by the CDDH technique.

The tubes with the remaining 50 µL of bacterial suspension were stored frozen at **−**20°C for further analysis by the limulus amebocyte lysate assay (PYROGENT™-5000; Lonza, Walkersville, Maryland, USA). As an additional control, endotoxin was quantified in 10 mini-implants removed from their original packages and not used to verify whether contamination occurred during manufacturing and/or packaging.

The presence of 40 bacterial species grouped according to the microbial complexes described by Socransky, et al.^13^ (1994) (Actinomyces group, purple, yellow, green, orange, red complexes and other species) was assessed in each sample using the CDDH technique^14^.

The amount of bacterial endotoxin on the mini-implants, expressed in EU/mL (endotoxin units *per* milliliter) was quantified by the limulus amebocyte lysate PYROGENT™ 5000 (Lonza, Walkersville, Maryland, USA), following the manufacturer’s instructions^12^.

### Statistical analysis

A comparative analysis of the patients’ sex and age and the mean time of permanence of the mini-implants in the mouth was performed by the test of difference of means for continuous variables and test of difference of proportions (Wald test) for categorical variables, considering the individuals as clusters. The other results were analyzed by the nonparametric Wilcoxon rank-sum test considering the clusters^15^ followed by a False Discovery Rate (FDR)^16^ to adjust for multiple comparisons. All analyses were performed using the SAS (Statistical Analysis System) software for Windows version 9.3 (SAS Institute, Inc., Cary, North Carolina, USA). The significance level was set at 5%.

## Results

The descriptive analysis of patient data showed no statistically significant difference between groups of successful and failed mini-implants with respect to sex and age. Only the mean time of permanence in the mouth of the mini-implants presented significant difference between groups (p<0.05).

**Figure 1:**
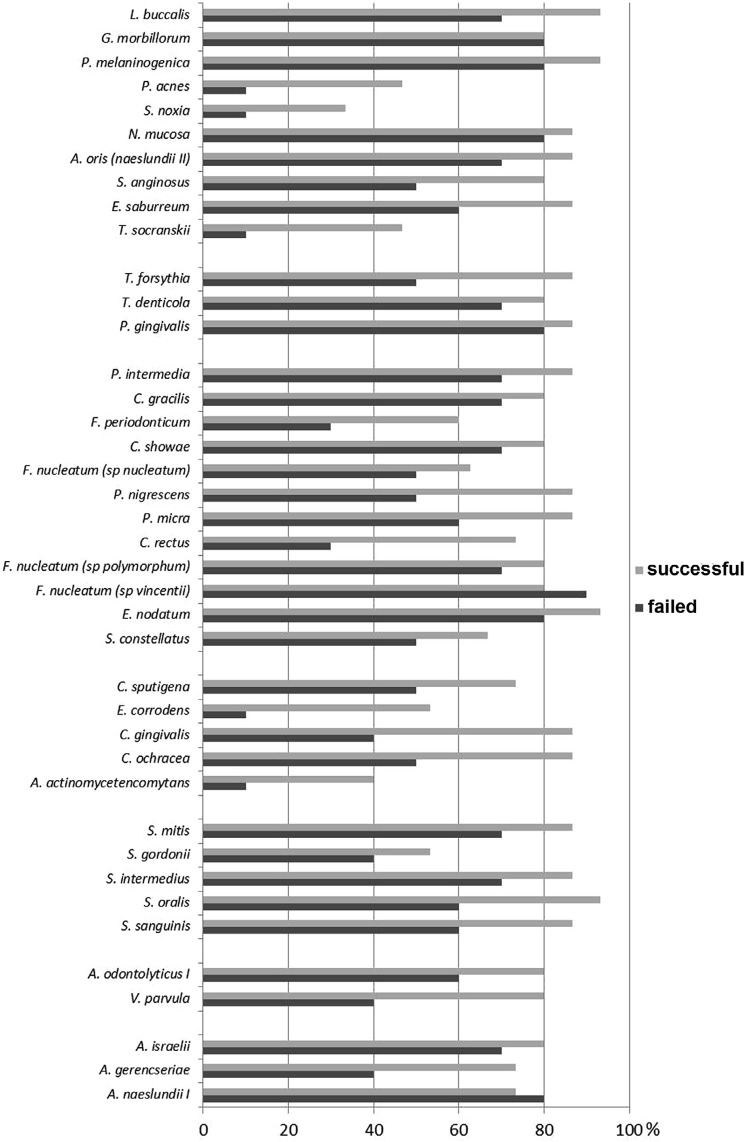
Percentage of occurrence of the 40 microbial species in successful and failed mini-implants

All 40 microbial species of the Actinomyces group, purple, yellow, green, orange, red complexes and other species (100%) were observed in both groups, although with different frequencies (Figure 1). No significant difference (p=0.2824) was found between successful and failed mini-implants considering the frequency of the microbial complexes.

Regarding the semi-quantitative analysis (bacterial cell count), the median of number of microorganisms of the 40 species in the group of successful and failed mini-implants was 12,950,000 and 8,490,000, respectively. No significant difference was found between the groups regarding the total number of microorganisms (p=0.75480). Considering the bacterial species alone, although there was an increase for *P. micra, T. denticola* and *E. saburreum,* it was not statistically significant (p>0.05), after adjusting for multiple comparison test (FDR) (Table 1). No significant difference was observed among the bacterial complexes in the semi-quantitative analysis (Table 2).

**Table 1:** Detection of microorganisms in successful and failed mini-implants (MI)

Microorganisms	M(Q1-Q3) Successful MI n=15	M(Q1-Q3) Failed MI n=10	Z	p	p (FDR)†
Actinomyces group					
*A. naeslundii I (12104a)*	100,000 (0 – 500,000)	500,000 (10,000 – 1,000,000)	0.9863	0.3239	0.6491
*A. gerencseriae (23860a)*	10,000 (0 – 100,000)	0 (0 – 500,000)	-0.0069	0.9945	0.9945
*A. israelli (12102a)*	500,000 (100,000 – 500,000)	500,000 (0 – 500,000)	-0.1552	0.8767	0.9228
*A. oris (naeslundii II) (43146a)*	500,000 (10,000 - 500,000)	55,000 (0 - 100,000)	-12.730	0.2030	0.6491
Purple Complex					
*V. parvula (10790a)*	500,000 (100,000 – 1,000,000)	0 (0 – 500,000)	-0.9144	0.3605	0.6555
*A. odontolyticus I (17929a)*	500,000 (10,000 – 500,000)	55,000 (0 – 500,000)	-14.236	0.1546	0.6491
Yellow Complex					
*S. sanguinis (10556a)*	500,000 (100,000 – 1,000,000)	55,000 (0 – 500,000)	-15.493	0.1213	0.6491
*S. oralis (35037a)*	500,000 (100,000 – 1,000,000)	100,000 (0 – 500,000)	-11.029	0.2701	0.6491
*S. intermedius (27335a)*	500,000 (100,000 – 500,000)	100,000 (0 – 500,000)	-0.3049	0.7604	0.8471
*S. gordonii (10558a)*	10,000 (0 – 500,000)	0 (0 – 500,000)	-0.3024	0.7624	0.8471
*S. mitis (49456a)*	500,000 (100,000 – 1,000,000)	300,000 (0 – 500,000)	-0.4891	0.6247	0.8471
Green Complex					
*A. actinomycetemcomitans (43718a + 29523a)*	0 (0 – 500,000)	0 (0 - 0)	-0.4299	0.6673	0.8471
*C. ochracea (33596a)*	100,000 (100,000 – 500,000)	50,000 (0 – 100,000)	-0.9559	0.3391	0.6491
*C. gingivalis (33624a)*	100,000 (10,000 – 500,000)	0 (0 – 100,000)	-10.409	0.2979	0.6491
*E. corrodens (23834a)*	10,000 (0 – 100,000)	0 (0 - 0)	-0.7213	0.4708	0.6975
*C. sputigena (33612a)*	500,000 (0 – 1,000,000)	5,000 (0 – 100,000)	-11.204	0.2625	0.6491
Orange Complex					
*S. constellatus (27823a)*	10,000 (0 - 500,000)	5,000 (0 – 100,000)	-0.4025	0.6873	0.8471
*E. nodatum (33099a)*	500,000 (100,000 – 500,000)	300,000 (10,000 – 500,000)	-0.0882	0.9298	0.9536
*F. nucleatum (sp vincentii) (49256a)*	100,000 (10,000 – 500,000)	100,000 (100,000 – 500,000)	0.4375	0.6617	0.8471
*F. nucleatum (sp polymorphum) (10953a)*	500,000 (100,000 – 1,000,000)	100,000 (0 – 500,000)	-0.2265	0.8208	0.8874
*F. nucleatum (sp nucleatum) (25586a)*	500,00 (0 -500,000)	50,00 (0 – 500,000)	-0.8046	0.4210	0.6736
*C. rectus (33238a)*	100,000 (0 – 100,000)	0 (0 – 10,000)	-17.385	0.0821	0.6491
*P. micra (33270a)*	500,000 (500,000 – 1,000,000)	100,000 (0 – 100,000)	-2,2159	0.0267*	0.5340
*P. nigrescens (33563a)*	100,000 (10,000 – 500,000)	50,000 (0 – 500,000)	-11.087	0.2676	0.6491
*C. showae (51146a)*	100,000 (10,000 – 500,000)	10,000 (0 – 10,000)	-0.8467	0.3972	0.6620
*F. periodonticum (33693a)*	100,000 (0 – 500,000)	0 (0 – 10,000)	-0.9821	0.3261	0.6491
*C. gracilis (33236a)*	100,000 (10,000 – 500,000)	10,000 (0 – 100,000)	-0.3369	0.7362	0.8471
*P. intermedia (25611a)*	500,000 (100,000 – 1,000,000)	55,000 (0 – 500,000)	-14.973	0.1343	0.6491
Red Complex					
*P. gingivalis (33277a)*	500,000 (10,000 – 1,000,000)	500,000 (10,000 – 500,000)	-0.7384	0.4603	0.6975
*T. denticola (B1b)*	500,000 (500,000 – 500,000)	100,000 (0 – 100,000)	-2,7199	0.0065*	0.2600
*T. forsythia (43037a)*	500,000 (100,000 – 1,000,000)	5,000 (0 – 500,000)	-13.922	0.1639	0.6491
Other species					
*T. socranskii (S1b)*	0 (0 – 10,000)	0 (0 - 0)	-0.8784	0.3797	0.6603
*E. saburreum (33271a)*	500,000 (100,000 – 500,000)	10,000 (0 – 100,000)	-19.712	0.0487*	0.6491
*S. anginosus (33397a)*	100,000 (10,000 – 500,000)	5,000 (0 – 500,000)	-11.209	0.2623	0.6491
*N. mucosa (19696a)*	1,000,000 (100,000 – 1,000,000)	100,000 (10,000 – 500,000)	-16.893	0.0912	0.6491
*S. noxia (43541a)*	0 (0 – 100,000)	0 (0 - 0)	-0.3673	0.7134	0.8471
*P. acnes (11827a + 11828a)*	0 (0 – 10,000)	0 (0 - 0)	-13.419	0.1796	0.6491
*P. melaninogenica (25845a)*	500,000 (100,000 – 1,000,000)	100,000 (100,000 – 1,000,000)	-10.475	0.2949	0.6491
*G. morbillorum (27824a)*	500,000 (10,000 – 500,000)	10,000 (10,000 – 100,000)	-0.9525	0.3408	0.6491
*L. buccalis (14201a)*	500,000 (100,000 – 500,000)	100,000 (0 – 500,000)	-0.5614	0.5746	0.8209

a: ATCC – American Type Culture Collection

b: Forsyth Institute, Boston, MA

*: p-value statistically significant for the Wilcoxon rank-sum test considering the conglomerate

Z: statistics value for the Wilcoxon rank-sum test considering the conglomerate

Values are expressed as M(Q1-Q3), where M is the median, Q1 is the first quartile and Q3 is the third quartile

†FDR: False Discovery Rate to adjust for multiple comparisons

**Table 2:** Results of the semi-quantitative analysis for detection of microorganisms in successful and failed mini-implants (MI) according to the bacterial complexes

Microorganisms	M(Q1-Q3) Successful MI n=15	M(Q1-Q3) Failed MI n=10	Z	p*
Blue Complex	610,000 (210,000 – 1,100,000)	1,050,000 (100,000 – 1,500,000)	0.6549	0.5125
Purple Complex	1,000,000 (500,000 – 1,500,000)	100,000 (0 – 1,000,000)	-12.923	0.1962
Yellow Complex	1,300,000 (700,000 – 3,500,000)	705,000 (100,000 – 2,100,000)	-12.531	0.2102
Green Complex	620,000 (120,000 – 2,600,000)	100,000 (0 – 700,000)	-12.803	0.2004
Orange Complex	3,340,000 (1,030,000 – 6,700,000)	1,570,000 (200,000 – 3,210,000)	-0.5771	0.5639
Red Complex	1,500,000 (1,100,000 – 2,000,000)	600,000 (210,000 – 1,100,000)	-18.198	0.0688
Other species	2,710,000 (1,500,000 – 4,210,000)	1,165,000 (300,000 – 2,200,000)	-13.123	0.1894

*: p-value for the Wilcoxon rank-sum test considering the conglomerate

Z: statistics value for the Wilcoxon rank-sum test considering the conglomerate

Values are expressed as M(Q1-Q3), where M is the median, Q1 is the first quartile and Q3 is the third quartile

The quantification of endotoxin revealed median values of 65,750 EU/mL for the successful mini-implants and 43,500 EU/mL for the failed mini-implants. No significant difference was found between the groups (p=0.63613) (Table 3).

**Table 3:** Quantification of endotoxin in successful and failed mini-implants (MI)

	M(Q1-Q3) Successful MI n=14	M(Q1-Q3) Failed MI n=9	Z	p*
Endotoxin units (EU)	65,750 (54,000 – 119,000)	43,500 (30,300 – 67,900)	-0.4731	0.6361

*: p-value for the Wilcoxon rank-sum test considering the conglomerate

Z: Z statistics value for the Wilcoxon rank-sum test considering the conglomerate

Values are expressed as M(Q1-Q3), where M is the median, Q1 is the first quartile and Q3 is the third quartile

Bacterial endotoxin was not detected in the group of mini-implants examined immediately after removal from their packages and not used clinically (values <0.01 EU).

## Discussion

Although the chronic inflammation caused by retention of bacterial biofilm has been implicated in the excessive mobility and consequent loss of mini-implants^6^, few studies have investigated microbial contamination around mini-implants used as temporary orthodontic anchorage devices^4^ ^,^ ^6^ ^,^ ^7^.

Previous studies using microbial culture techniques, polymerase chain reaction (PCR) and microarray^4^ ^,^ ^6^ ^,^ ^7^ have identified periodontopathogenic microorganisms in the peri-implant sulci or surfaces of mini-implants. This study evaluated the contamination of mini-implant surfaces using checkerboard DNA-DNA hybridization, as this biomolecular technique can detect, in a single analysis, 40 microbial species, including the Actinomyces group, purple, yellow, green, orange, red complexes and other species. It has been used in Orthodontics to evaluate the contamination of metallic and ceramic brackets^12^ ^,^ ^17^ ^-^ ^19^ and the subgingival microbiota in patients undergoing orthodontic treatment^20^.

In this study there was no significant difference in the frequency of complexes between the groups. In a previous study^6^, bacterial samples collected from the peri-implant sulcus surrounding had the 8 failed and 4 successful mini-implants subjected to a universal bacteria-directed real-time quantitative PCR for quantification in combination with a microarray-based identification of 20 selected species. *A. odontolyticus* and *V. parvula* (both from purple complex) and *S. gordonii* and *S. mitis* (both from the yellow complex) were detected in 100% of the samples of both groups. *S. constellatus* (orange complex), *P. gingivalis* (red complex) and *A. actinomycetemcomitans* (green complex) were not detected in either of the groups. These findings differ from those of the present study in which all 40 microbial species were detected in both mini-implant groups. Despite the methodological differences, Apel, et al.^6^ (2009) observed no differences between groups of successful and failed mini-implants regarding the total number of microorganisms or the microbial composition, and were unable to identify a specific aggressive microbiota in the failed mini-implants.

Tortamano, et al.^7^ (2012) also used PCR to assess 3 periodontopathogenic bacteria on the surfaces of 15 unstable and 16 stable mini-implants and reported very similar results to those of the present study, with a higher incidence of microorganisms in the stable mini-implants. In addition, they found no association between periodontopathogenic microorganisms and loss of stability.

It should be mentioned that neither of those studies^6^ ^,^ ^7^ performed a semi-quantitative analysis of each individual microorganism, making it difficult to establish a proper comparison with the present results. In this study, the semi-quantitative analysis of the microorganisms revealed no significant difference between groups for all species.

According to Lindhe and Meyle^21^ (2008), the inflammation associated with prosthetic implants due to poor oral hygiene may cause peri-implantitis, which starts in the soft tissue and extends slowly along the screw, causing mobility and consequently loss of the implant. As the progression of peri-implantitis and chronic periodontitis usually is slow and may take several years, inflammation of the gingival tissues around mini-implants might not be determinant for clinical success or failure, considering the short period of these devices in the mouth, especially in failed cases. These results agree with those of Apel, et al.^6^ (2009) and Tortamano, et al.^7^ (2012), who found no significant differences in the detection of microorganisms in mini-implants with and without stability, making bacterial contamination not determinant in this process.

The results of this study could be associated with the longer time of successful mini-implants in the mouth compared with the failed ones. Randomized clinical trials should be conducted to determine how the microbiota is established in both situations.

It is known that endotoxin has high affinity for several materials, including titanium^22^, and that contamination with endotoxin causes the loss of orthopedic implants, inhibits initial osseointegration and induces cytokine production and osteoclast differentiation^23^ ^-^ ^25^. Endotoxin also plays an important role in the development of chronic periodontitis, possibly affecting the healing process and inflammation, with reduction of cell proliferation^22^, and is associated with pathologies involving dental implants, including osseointegration failure and development of peri-implantitis^11^ ^,^ ^22^. Additionally, recent *in vitro* studies confirmed the effect of endotoxin on the induction of genic expression of proinflammatory cytokines^11^ and bone resorption around contaminated prosthetic implants in an animal model^26^. Following the same reasoning, it could also occur with mini-implants.

The contamination of mini-implants by Gram-negative periodontopathogenic bacteria has been demonstrated^6^ ^,^ ^7^. However, to the best of our knowledge, this is the first study to evaluate the contamination of these devices by endotoxin, and thus comparison of results is not possible.

Considering the importance of endotoxin in inflammation and bone resorption, the amount of endotoxin in well-retained and unstable mini-implants was quantified in this study to assess whether contamination by endotoxin, rather than implant stability, would be a determinant factor for clinical success.

The affinity of endotoxin for metallic materials has been demonstrated in two studies^12^ ^,^ ^27^ that detected it on the surfaces of orthodontic brackets. The authors considered endotoxin contamination as a probable cause of the gingival inflammation commonly observed in patients undergoing orthodontic treatment, as the placement of brackets close to the gingival sulcus might affect endotoxin concentration, predisposing the periodontal tissues to inflammation. According to these results, both successful and failed mini-implants of both groups were heavily contaminated with endotoxin, justifying, in part, the occurrence of peri-implant soft tissue inflammation reported in clinical investigations^4^ ^,^ ^6^ ^,^ ^7^, as the mini-implants are in intimate contact with the periodontal tissues. However, no significant difference was found between successful and failed mini-implants in this study.

Regarding the additional controls, the mini-implants examined immediately after removal from their packages and not used in the patients were free of endotoxin (<0.01 EU). As the acceptable level of endotoxin medical-hospital products is <0.5 EU^28^, the amount of endotoxin detected in the mini-implants of this study originated from oral bacteria that colonized the surfaces of these devices.

Nelson-Filho, et al.^12^ (2011) detected endotoxin in amounts ranging from 0.09136 to >1.9000 EU/mL (median=0.6673 EU/mL) on the surface of orthodontic brackets after 30 days in the mouth. Therefore, comparison of endotoxin quantification on the surfaces of brackets, successful (median=65,750 EU/mL) and failed (median=43,500 EU/mL) mini-implants revealed a substantially greater amount of endotoxin on the bone anchorage devices. This fact has a clinical relevance because the endotoxin in contact with soft and mineralized tissues may act as a potent inductor of inflammation and bone resorption^12^, reinforcing the need for rigorous oral hygiene in orthodontic patients with mini-implants. In this study, significant gingival inflammatory alterations were observed in the patients of both groups. Additionally, the medians of endotoxin detected in the failed mini-implants could be explained by the shorter time of permanence in the mouth (6.7 months) compared with the successful implants (23.1 months).

In conclusion, in the studied population, it is possible that bacterial contamination and endotoxin on the mini-implants were not determinant for their loss of stability, and other factors related to orthodontic mechanics, mini-implant location and surgical technique would be more directly involved in the early loss of mini-implants. Further *in vivo* studies should be conducted to elucidate the participation of these factors on the success or early loss of these orthodontic anchorage devices.
